# New insight into the dynamic properties and the active site architecture of H-Ras p21 revealed by X-ray crystallography at very high resolution

**DOI:** 10.1186/1472-6807-10-38

**Published:** 2010-10-25

**Authors:** Björn U Klink, Axel J Scheidig

**Affiliations:** 1 Department of Biophysics, Division of Structural Biology, Saarland University, D-66421 Homburg/Saar, Germany; 2 Zoological Institute, Department of Structural Biology, Christian-Albrechts University of Kiel, Am Botanischen Garten 1-9, D-24118 Kiel, Germany; 3 Division of Structural Biology, Helmholtz Center for Infection Research, Inhoffenstraße 7, D-38124 Braunschweig, Germany

## Abstract

**Background:**

In kinetic crystallography, the usually static method of X-ray diffraction is expanded to allow time-resolved analysis of conformational rearrangements in protein structures. To achieve this, reactions have to be triggered within the protein crystals of interest, and optical spectroscopy can be used to monitor the reaction state. For this approach, a modified form of H-Ras p21 was designed which allows reaction initiation and fluorescence readout of the initiated GTPase reaction within the crystalline state. Rearrangements within the crystallized protein due to the progressing reaction and associated heterogeneity in the protein conformations have to be considered in the subsequent refinement processes.

**Results:**

X-ray diffraction experiments on H-Ras p21 in different states along the reaction pathway provide detailed information about the kinetics and mechanism of the GTPase reaction. In addition, a very high data quality of up to 1.0 Å resolution allowed distinguishing two discrete subconformations of H-Ras p21, expanding the knowledge about the intrinsic flexibility of Ras-like proteins, which is important for their function. In a complex of H-Ras•GppNHp (guanosine-5'-(β,γ-imido)-triphosphate), a second Mg^2+ ^ion was found to be coordinated to the γ-phosphate group of GppNHp, which positions the hydrolytically active water molecule very close to the attacked γ-phosphorous atom.

**Conclusion:**

For the structural analysis of very high-resolution data we have used a new 'two-chain-isotropic-refinement' strategy. This refinement provides an alternative and easy to interpret strategy to reflect the conformational variability within crystal structures of biological macromolecules. The presented fluorescent form of H-Ras p21 will be advantageous for fluorescence studies on H-Ras p21 in which the use of fluorescent nucleotides is not feasible.

## Background

H-Ras p21 is a small guanosine nucleotide binding protein with low GTPase activity. It is involved in a variety of intracellular signaling pathways where it functions as a molecular switch by cycling between an active GTP-bound state and an inactive GDP-bound state. In the active state, Ras binds different effector molecules like Raf, RalGDS and PI3K [1] (and references therein). Switching between the GTP and GDP-bound state involves conformational changes that are mainly located in two regions of the protein, which are called switch I and switch II. Since the energy barrier for the conversion between different conformations in these regions is relatively low, the protein can exist in sub-states that are in dynamic equilibrium with each other. ^1^H- and ^31^P-NMR spectroscopy experiments have shown that at least two conformational states are present in H-Ras p21 bound to the non-hydrolyzable GTP analogs guanosine-5'-(β,γ-imido)-triphosphate (GppNHp) or guanosine-5'-(β,γ-methylene)-triphosphate (GppCH_2_p) [2-4], even in the crystalline state [5,6]. In X-ray diffraction studies, these heterogeneities lead to electron density distributions for the flexible switch regions that are weak and/or difficult to interpret. As the dynamic properties of H-Ras p21 are very important for the binding of different nucleotides and for the interaction with various effector molecules, a more detailed understanding of the dynamics is crucial for understanding the functionality of this protein.

In this study, we present the results of high-resolution X-ray crystallographic investigations of a fluorescent form of truncated H-Ras p21 in complex with different nucleotides. This modified protein was generated to fit the needs of kinetic crystallography, i.e. initiation of the GTP hydrolysis reaction by flash photolysis of the GTP precursor 'caged GTP' and monitoring of the reaction by a covalently attached fluorophore. The development and applications of the FLUMIX fluorescence spectrometer which was used for kinetic X-ray crystallographic studies on this protein variant were described in detail by Klink et al. [7]. To complement that work, the obtained structural information about the modified protein will be discussed in detail in this work. Several crystal structures of the modified H-Ras p21 in different states reveal additional information about its dynamic properties and potentially provide new insights into the mechanism of GTP hydrolysis. Since similar dynamical properties of the switch regions are also observed in other Ras-like GTPases [8], the presented studies are of general significance for the Ras superfamily including Rho/Rac, Rab, Ran and Arf proteins, among others.

## Methods

### Expression and purification

A ptac expression plasmid [9] coding for a truncated form (aa 1-166) of wild-type H-Ras p21 (referred to as p21(wt)) with two modifications (Y32C, C118S) was transformed into the *Escherichia coli *(*E. coli) *strain CK600K, which is the strain K12 wild type CK600 containing the plasmid pDMI,1 [10]. Cells were grown in 10 l Standard I-medium (Merk, Germany, Ref. 107881) (50 μg/ml ampicillin, 50 μg/ml kanamycin) at 37°C. Recombinant protein expression was induced with 0.5 mM IPTG at OD_600_~0.7. The cells were harvested thirteen hours after induction at 25°C, washed with STE buffer (20 mM tris-(hydroxymethyl)-aminoethane (TRIS) adjusted with HCl to pH 7.5, 1 mM EDTA, 150 mM NaCl) and resuspended in lysis buffer (32 mM TRIS/HCl pH 7.6, 0.5 mM NaN_3_, 5 mM MgCl_2_, 5 mM dithiothreitol (DTT), 2 mM PMSF, 2 mM benzamidine). Cells were disrupted using a microfluidizer (Microfluidics, MA, USA) and centrifuged at 30,000 × g for 2 hours. The supernatant was loaded on a DEAE sepharose FF column equilibrated with buffer A (32 mM TRIS/HCl pH 7.6, 0.5 mM NaN_3_, 5 mM MgCl_2_, 5 mM DTT). After washing the column with buffer A containing 32 mM NaCl, the protein was eluted with buffer A containing 64 mM NaCl. Fractions containing H-Ras p21, as revealed by SDS PAGE, were pooled and concentrated to ~100 mg/ml with 10 kDa cutoff Amicon concentrator units (Millipore, USA). The concentrated protein was further purified and buffer-exchanged to buffer B (64 mM TRIS/HCl pH 7.6, 1 mM NaN_3_, 10 mM MgCl_2_, 5 mM DTT, 400 mM NaCl, 0.1 mM GDP) by gel-filtration chromatography on a Superdex 75 (26/60) column (GE Healthcare, UK).

### Chemical modification and nucleotide exchange

For fluorescence labeling on position C32, 1.5 ml of a 17 mg/ml protein solution in buffer C (75 mM K-phosphate pH 7.6, 5 mM MgCl_2_, 0.5 mM NaN_3_, 3 mM ascorbic acid/NaOH pH 7.0, 50 μM GDP) was labeled with *N*,*N*'-dimethyl-*N*-(iodoacetyl)-*N*'-(7-nitrobenz-2-oxa-1,3-diazol-4-yl)ethylenediamine ("IANBD amide"; Invitrogen, Germany, Ref. D-2004). For this purpose, 5 mg of the fluorophore were suspended in 130 μl DMSO using a supersonic bath, and the suspension was added in drops under continuous stirring to the protein solution. After one hour, this step was repeated with an additional 6.5 mg of fluorophore in 130 μl DMSO. During the reaction, the labeling efficiency was controlled by MALDI-TOF mass spectroscopy. After ~6 hours reaction time, the labeled protein was buffer-exchanged to buffer B, and unreacted fluorophore was removed via a PD10 desalting column (GE Healthcare, UK). The obtained protein solution was further purified by gel-filtration chromatography on a Superdex 75 (26/60) column (GE Healthcare, UK). ESI mass spectroscopy on the final protein solution showed no analyzable amounts of unlabeled or double-labeled protein. The proper labeling of the C32 position was verified by a combination of chemical digestion with 2-nitro-5-thiocyano-benzoic acid (NTCB) [11] and MALDI-TOF mass spectroscopy. Nucleotide exchange was performed as described [12], and was verified by HPLC analysis. The C32-IANBD fluorescently labeled form of H-Ras p21 (aa 1-166, Y32C, C118S) is referred to as p21(mod) throughout the text.

### Crystallization and treatment of crystals

Crystallization was performed at 18°C using the hanging-drop technique in 24-well Linbro plates (ICN, Germany). Crystallization droplets consisted of equal amounts of protein solution mixed with reservoir solution. The protein solution consisted of 64 mM TRIS/HCl pH 7.6, 20 mM MgCl_2_, 10 mM DTT, 0.1 mM NaN_3_, and varying concentrations of protein (see Table 1). The reservoir compositions for seven crystals that will be discussed in this work are listed in Table 1.

**Table 1:** Crystallization parameters for crystals C1 - C7.

crystal	nucleotide	c(protein) (mg/ml)	reservoir solution	cryo solution	reservoir volume	drop size	seeding technique	crystal age
**C1**	GppNHp	12.64	64 mM TRIS pH 7.6 20 mM MgCl_2 _10 mM DTT 0.1 mM NaN_3 _26% PEG 400	directly frozen	450 μl	2 μl	-	2 days

**C2**	GDP	17.27	64 mM TRIS pH 7.6 20 mM MgCl_2 _10 mM DTT 0.1 mM NaN_3 _35% PEG 400	directly frozen	500 μl	4 μl	-	6 months

**C3**	S-*caged *GTP	15.40	0.2 M Mg acetate 0.1 M HEPES pH 7.4 17% PEG 8000	XC-17/20 ^**a**^	1 ml	20 μl	*streak-seeding*	3 days

**C4**	R-*caged *GTP	13.35	0.2 mM Mg acetate 0.1 M HEPES pH 7.2 16% PEG 8000	~3 min in XC-16/10 ^**b**^, then transferred to XC-17/20 ^**a**^	500 μl	4 μl	*streak-seeding*	3 days

**C5**	R-*caged *GTP→GTP	13.35	0.2 M Mg acetate 0.1 M HEPES pH 7.2 16% PEG 8000	XC-17/20 ^**a**^	1 ml	4 μl	*streak-seeding*	3 days + ~4 min after photolysis

**C6**	R-*caged *GTP→GDP	13.35	0.2 M Mg acetate 0.1 M HEPES pH 7.2 16% PEG 8000	XC-17/20 ^**a**^	1 ml	4 μl	*streak-seeding*	3 days + 68 hours after photolysis

**C7**	R-*caged *GTP→GTP	13.35	0.2 M Mg acetate 0.1 M HEPES pH 7.2 16% PEG 8000	XC-17/20 ^**a**^	1 ml	4 μl	*streak-seeding*	3 days + ~3 min after photolysis

^a ^The composition of XC-17/20 cryo solution is 100 mM HEPES pH 7.2, 64 mM TRIS pH 7.6, 20 mM MgCl_2_, 200 mM Mg-acetate, 0,1 mM NaN_3_, 17% PEG 8000 (freshly prepared), 20% glycerol.

^b ^The composition of XC-16/10 cryo solution is 100 mM HEPES pH 7.2, 64 mM TRIS pH 7.6, 20 mM MgCl_2_, 200 mM Mg-acetate, 0,1 mM NaN_3_, 16% PEG 8000 (freshly prepared), 10% glycerol.

Depending on the required cryo-protectant solution and/or crystal state, the analyzed crystals were treated differently prior to flash-cooling in liquid nitrogen (see Table 1). Crystals C1 and C2 could be flash-cooled in liquid nitrogen without further manipulation. Crystals C3 and C4 were soaked for several minutes in a stabilizing cryo-protectant solution prior to flash-cooling. Crystals C5 - C7 were transferred to a cryo-protectant solution, and the enzymatic reaction in the protein crystal was triggered by photolysis of the caged nucleotide with UV light from a HeCd-laser (Soliton, Germany, Model IK5652R-G). Crystals C5 and C7 were immediately flash-cooled after the nucleotide was completely photolyzed to GTP and 2-nitrosoacetophenone ("released cage group"), which was verified by fluorescence measurements.

### Data collection, structure determination and model analysis

X-ray diffraction data from crystals C1 - C7 were collected at the synchrotron beamlines ID14-1 and ID 14-4 (ESRF, Grenoble, France), and PX-I (SLS, Villigen, Switzerland), respectively, using monochromatic radiation with different wavelengths in the range of 0.827 Å to 0.976 Å (see Table 2). All data processing was performed using the program package XDS/XSCALE [13,14]. The structures were solved by molecular replacement with the program MOLREP [15], as implemented in the CCP4 program package [16]. The homology models were derived from H-Ras p21 in complex with GDP and GppNHp (PDB entry codes 4Q21[17] and 5P21[18], respectively), or from partially refined models of other datasets from the modified H-Ras p21. In all cases, a single molecular replacement solution with one molecule per asymmetric unit could be obtained. Refinement was performed using the program REFMAC5 as implemented in the CCP4 program package [16,19,20]. The model was checked and rebuilt against σ_A_-weighted electron density maps with the program O [21]. Omit maps were generated by using the randomized omit map procedure [22]. The coordinates of the questioned peptide regions were removed from the model and a random translation of <0.2 Å was added to each of the remaining coordinates. This altered model was subjected to 10 rounds of restrained refinement with REFMAC and omit electron density maps with coefficients 2F_obs_-1F_calc _were calculated. The program ACONIO [23] was used to separate protein models containing alternative conformations into two separate PDB-files for display and model rebuilding with O, and to merge the two files for refinement with REFMAC5. The individual statistics for data collection, processing and refinement are given in Table 2. Images were created using the program PyMOL, Version 0.97 [24].

**Table 2:** Data statistics for crystals C1 - C7.

	Crystal name and nucleotide content						
	**C1 **GppNHp	**C2 **GDP	**C3 **S-*caged *GTP	**C4 **R-*caged *GTP	**C5 **R-*caged *GTP→GTP	**C6 **R-*caged *GTP→GDP	**C7 **R-*caged *GTP→GTP

**Data collection and processing**							

**Synchrotron (Beamline)**	ESRF (ID14-1)	SLS (PX-I)	ESRF (ID14-4)	ESRF (ID14-4)	ESRF (ID14-4)	ESRF (ID14-4)	ESRF (ID14-4)

**Area detector**	Q4R ADSC	CCD CHESS	Q4R ADSC	Q4R ADSC	Q4R ADSC	Q4R ADSC	Q4R ADSC

**Temperature (K)**	100	100	100	100	100	100	100

**Wavelength (Å)**	0.934	0.827	0.920	0.939	0.976	0.939	0.976

**Space group**	R32	C222(1)	P4(1)	P4(1)	P4(1)	P4(1)	P4(1)

**Cell dimensions (Å)**	*a *= *b *= 88.5 *c *= 144.1	*a *= 49.0 *b *= 53.8 *c *= 116.0	*a = b = *69.1 *c *= 35.5	*a = b = *69.1 *c *= 35.5	*a = b = *69.3 *c *= 35.0	*a = b = *69.4 *c *= 34.8	*a = b = *69.3 *c *= 35.0

**Resolution limit**^**a **^**(Å)**	67.42 - 1.70 (1.75 - 1.70)	50.00 - 0.99 (1.00 - 0.99)	69.01 - 1.24 (1.26 - 1.24)	69.34 - 1.00 (1.05 - 1.00)	69.34 - 1.25 (1.30 - 1.25)	69.34 - 1.22 (1.30 - 1.22)	69.34 - 1.30 (1.40 - 1.30)

**Number of recorded reflections**	276701	400222	317470	541277	283084	286285	258379

**Number of unique recorded reflections**	23933	82821	46194	91096	46204	47013	41195

**Average redundancy**	11.6	4.8	6.9	5.9	6.1	6.1	6.3

***R***_***sym***_^**a,b **^**(%)**	8.1 (86.1)	4.9 (60.5)	4.9 (33.4)	5.2 (67.9)	4.3 (51.5)	4.6 (45.7)	5.5 (54.9)

**Average *I/σ***^**a **^**(I)**	16.9 (2.1)	15.0 (2.3)	18.7 (3.1)	13.6 (2.3)	20.7 (3.5)	21.5 (3.0)	16.05 (3.14)

***B*-factor from Wilson plot (Å**^**2**^**)**	30.9	12.0	18.3	14.0	21.1	22.1	22.6

**Refinement statistics**							

**Resolution range**^**a **^**(Å)**	67.42 - 1.80 (1.85 - 1.80)	50.00 - 1.00 (1.03 - 1.00)	69.01 - 1.24 (1.27 - 1.24)	69.34 - 1.05 (1.08 - 1.05)	69.34 - 1.25 (1.28 - 1.25)	69.34 - 1.22 (1.25 - 1.22)	69.34 - 1.30 (1.33 - 1.30)

**Number of unique reflections**	19368	76394	44152	78796	43911	44702	39138

**Completeness of data**^**a **^**(%)**	99.88 (100.00)	96.95 (96.03)	96.78 (76.25)	99.93 (99.97)	99.92 (99.94)	94.12 (55.47)	100.00 (100.00)

***R***_***work***_^**a,c **^**/*R***_***free***_^**a,d **^**(%)**	14.6/18.5 (18.9/23.7)	14.4/16.3 (27.0/27.7)	14.6/18.6 (21.8/21.2)	15.6/18.1 (26.5/27.9)	14.8/17.0 (24.6/24.3)	14.9/17.6 31.4/34.5	14.9/18.0 (27.2/30.9)

**Ramachandran plot**^**e **^**Favored, allowed, generous, disallowed (%)**	94.7, 5.3, 0.0, 0.0	89.3, 10.0, 0.7, 0.0	87.3, 10.3, 1.0, 0.3	89.3, 9.0, 1.0, 0.7	90.0, 9.7, 0.0, 0.3	90.0, 9.0, 0.7, 0.3	89.7, 10.0, 0.0, 0.3

**Rmsd on bond lengths (Å)**	0.016	0.012	0.012	0.015	0.015	0.011	0.015

**Rmsd on bond angles (deg)**	1.808	1.719	1.625	1.855	1.742	1.534	1.753

**Mean *B*-factors (Å**^**2**^**)**							

**Backbone (conformation A|B)**	30.0	8.0 | 8.4	14.2 | 14.9	5.2 | 4.8	7.0 | 8.5	18.0 | 16.6	13.0 | 14.9

**Side-chain (conformation A|B)**	34.4	10.3 | 11.0	16.1 | 17.2	7.4 | 6.9	10.1 | 11.3	20.58 | 18.3	15.9 | 17.7

**Nucleotide (conformation A|B)**	25.7	6.8 | 6.8	15.0 | 16.2	6.0 | 7.3	6.7 | 7.2	20.0 | 16.0	12.3 | 12.6

**Fluorophore (conformation A|B)**	85.6	32.0 | 33.4	23.6 | 30.8	19.7 | 12.5	42.0 | 32.2	-	43.5 | 42.4

**Solvent**	49.4	24.0	26.3	28.5	29.7	38.2	24.1

**PDB ID**	2CL0	2CE2	2CL6	2EVW	2CL7	2CLD	2CLC

^a ^Values in parentheses are for the high-resolution bin.

^b ^$R_{sym}=100\times \sum_{h}\sum_{i}\left|I_{i}\left(h\right)-\langle I_{i}\left(h\right)\rangle \right|/\sum_{h}\sum_{i}I_{i}\left(h\right)$, where *I*_*i*_*(h) *is the *i*th measurement and *Ii(h) *is the mean of all measurements of *I(h) *for Miller indices hkl.

^c ^$R=\sum \left(\left|\left|F_{obs}\right|-k\left|F_{calc}\right|\right|\right)/\sum \left|F_{obs}\right|$, where *F*_*obs *_and *F*_*calc *_are observed and calculated structure factor amplitudes, respectively.

^d ^*R*_*free *_value is the *R *value obtained for a test set of reflections, consisting of a randomly selected 5% subset of the diffraction data not used during refinement [36].

^e ^Calculated using the program PROCHECK [37].

### The 'two-chain-isotropic-refinement' strategy for interpretation of alternative main-chain conformations

With the high resolution available for most of the analyzed datasets, it became obvious in early steps of refinement that several residues occupy at least two alternative conformations. In the first rounds of refinement only those alternative conformations were built which displayed significant differences in the main-chain or side-chain trace (~25% of all residues for crystal C4 (p21(mod)•R-caged GTP)). In a small globular protein like H-Ras p21, it is likely that this number and extent of alternative conformations affect most of the remaining residues of the protein to some degree. Therefore, in the last rounds of refinement, two alternative conformations for all remaining residues in the refinement were included, such that the whole protein molecule was modeled with two conformations. A constant occupancy of 0.5 for both conformations was kept for the whole protein chain, as the electron density did not provide evidence for a significant deviation from an equal contribution of both chains. We will attribute this procedure as the 'two-chain-isotropic-refinement' strategy throughout the rest of this work. For crystals C2 - C7, an additional significant reduction of both *R*_*work *_and *R*_*free *_by more than 1% and a significant improvement of the electron density was observed.

As an alternative to the interpretation with two protein chains and individual *B*-factor refinement, anisotropic *B*-factor refinement for the whole protein was tested, using alternative conformations only where clearly visible. However, by using anisotropic *B*-factors the drop in the *R*_*work *_as well as the *R*_*free *_was not as significant as in the 'two-chain-isotropic-refinement' strategy. Since the overall increase of parameters per atom is higher with anisotropic *B*-factors compared to the 'two-chain-isotropic-refinement', the latter strategy provided the better observation/parameter ratio. Even of more importance was the more straightforward interpretation of the final structure. The use of two conformations for the whole protein chain provides information about two extreme conformations of the protein, which is much more intuitive and informative than a manual analysis of the more abstract anisotropic *B*-factors. One further benefit within Refmac is that a strict separation of all atoms in two conformations lowers the risk of misassigning atoms to a wrong chain, which would lead to unrealistic repulsions as Refmac only considers interactions between atoms with the same chain identifier. Such unrealistic interactions can be of a problem if alternative conformations are wrongly assigned in conventional refinement strategies. In the 'two-chain-isotropic-refinement' strategy, the decision to interpret a crystal structure either by two alternative conformations or by the use of anisotropic *B*-factors mostly depends on the question if the data quality allows adequate (manual) interpretation of both conformations. In this respect, the method differs from the 'ensemble refinement' strategy as described by Levin et al. 2007 [25], which is based on automated refinement of multiple identical copies of the same protein conformation. As discussed by Levin et al. [25], ensemble refinement can be applied to describe small divergences of different protein subpopulations even in data with relatively low resolution. However, as it does not utilize manual building of each diverging chain, it has a far lower radius of convergence than the 'two-chain-isotropic-refinement' method and would not be useful to describe large-scale heterogeneities as observed in p21(mod).

## Results and Discussion

### Crystal structures of H-Ras p21 in different states

**Figure 1:**
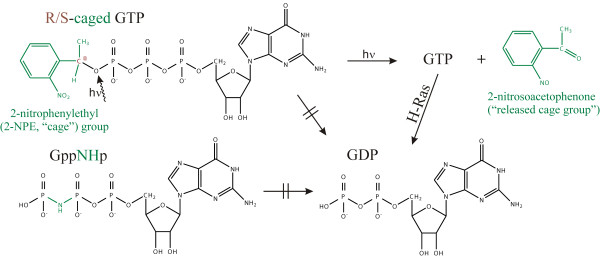
**Schematic representation of different nucleotides and relevant photolysis and hydrolysis reactions**. For the non-hydrolyzable GTP analogs caged GTP and GppNHp, the modifications compared to GTP are marked in green. The chiral carbon atom leading to the diastereomers R- and S-caged GTP is marked in brown.

For kinetic crystallography experiments, a truncated H-Ras p21 (aa 1-166) was modified by substituting Tyr32 and Cys118 with cysteine and serine, respectively, and covalently attaching an NBD fluorophore to Cys32 ("p21(mod)"). The fluorophore was used to monitor the protein state after photolysis of p21(mod) crystals complexed with R- or S-caged GTP. In this way, completion of light-induced GTP release and H-Ras-catalyzed hydrolysis of GTP to GDP could be analyzed by fluorescence measurements using our newly developed FLUMIX spectrometer [7]. The reactions triggered by caged GTP photolysis and the different nucleotides used in this work are summarized in Figure 1. Seven crystal structures of p21(mod) complexed with different nucleotides were analyzed (crystallization/photolysis parameters and data statistics are summarized in Tables 1, 2):

p21(mod) was cocrystallized with the non-hydrolyzable GTP analog GppNHp (crystal structure C1), GDP (crystal structure C2) and with the light-inducible GTP precursors R- and S-caged GTP (crystal structures C3 and C4). Additionally, crystals complexed with R-caged GTP were photolyzed to induce *in crystallo *GTP release. Crystals C5 and C7 were incubated for 3-4 minutes after photolysis, which did not allow significant hydrolysis of the formed GTP nucleotide, but was long enough for completion of conformational changes due to the release of 2-nitrosoacetophenone ("released cage group"), as analyzed by a change in the fluorescence signal (described in detail by Klink et al. [7]). Crystals C5 and C7 were photolyzed in two different experimental setups, as discussed in detail by Klink et al. [7]. Fluorescence experiments showed that in a hanging drop crystal setup, the released cage group almost completely diffused out of the active site (crystal C5), while in crystal C7 (photolyzed in a humid gas stream without significant volumes of mother liquor around the crystal) it was still partially present in the active site. Therefore, only crystal C5 will be further discussed in this work. Another p21(mod)•R-caged GTP crystal was photolyzed and incubated for ~ 3 days to allow complete *in crystallo *hydrolysis of the formed GTP nucleotide to GDP (crystal structure C6).

### Structure and activity compared with wild-type H-Ras p21

**Figure 2:**
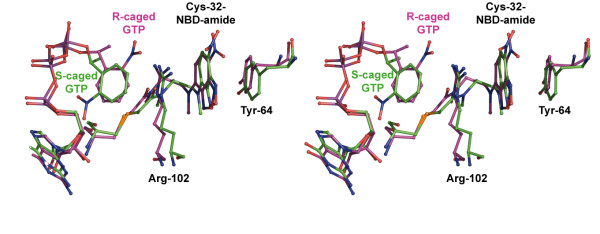
**Structural environment of the fluorophore and caged GTP in p21(mod)**. Stereo representation of the structural environment for the NBD fluorophore and the 2-nitrophenylethyl group of caged GTP in p21(mod)•R-caged GTP (magenta sticks) and p21(mod)•S-caged GTP (green sticks). The aromatic moieties form a strong cation-π/π - π stacking interaction with two residues from a neighboring molecule (Arg102 and Tyr64). For a better representation, only one sub-conformation of each structure is shown.

The overall G-domain fold, which is common for essentially all guanosine nucleotide binding proteins [26,27], was confirmed by all analyzed structures of the modified H-Ras p21. Small but significant differences to the wild-type protein (e.g. [17,18]) were localized mainly in the flexible loops L2 (residues 30-36) and L4 (residues 60-66), and in residues contacting these regions. In p21(mod) in complex with R-caged GTP, loop L4 (residues 60-66) is restrained in an unnatural conformation by cation-π/π - π stacking interactions between the 2-nitrophenylethyl (2-NPE, "cage") group of the nucleotide, the fluorophore which is attached to residue Cys32, and Arg102 and Tyr64 from a neighboring molecule (Figure 2). This artificial restraint is eliminated upon photolysis of caged GTP, since the cage group which is crucial for this stacking interaction is released upon photolysis.

Surprisingly, the structure of p21(mod) in complex with GppNHp (crystal C1) displays an unexpected second Mg^2+ ^ion coordinating the γ-phosphate of the nucleotide and one oxygen of the carboxyl-group of Glu63. This finding might have important consequences for a more detailed understanding of the GTP hydrolysis mechanism in general (see below).

The modified protein has a reduced intrinsic GTP hydrolysis rate (t_½ _= 430 ± 18 min at 21°C [7] compared to t_½ _= 18.7 min at 37°C for the wild type protein [10]), probably due to the restricted geometry of the switch regions. Large conformational heterogeneities in these regions could be interpreted with the 'two-chain-isotropic-refinement' strategy (see Materials and methods). In this way, a detailed analysis of the conformational dynamics of the flexible loops and regions in proximity to those, like helix α2 (residues 67-74) and helix α3 to loop L7 (residues 98-108), becomes possible.

### Catalysis in the crystalline state does not require identical conformational changes as observed in solution

With a resolution of 1.0 Å, the data quality of p21(mod) cocrystallized with GDP is exceptionally high and to our knowledge represents the highest resolution for a G-protein described to date. Virtually all residues are well defined in the observed electron density. The structure is essentially identical to the structure of the wild-type protein (PDB entry code 4Q21; [17]), confirming that a native protein conformation is preserved in the modified protein. Particularly, the attachment of an NBD fluorophore to Cys32 does not restrict the conformation of loop L2, which occupies an almost identical conformation as in the GDP-bound wild-type protein. The fluorophore itself occupies a weakly defined position and is most likely involved in π-stacking interactions with Tyr40. This contact is analogous to the wild-type protein, where Tyr32 forms a side-chain hydrogen bond with Tyr40.

After confirming a native protein fold, we analyzed how the p21(mod) crystal structure obtained by photolysis of caged GTP and subsequent *in crystallo *hydrolysis of the released GTP to GDP (crystal C6) compares to the analogous structure obtained by cocrystallization with GDP (crystal C2). The electron density around the nucleotide in the 'photolyzed' structure C6 is well defined, and verifies complete hydrolysis to GDP. Thr35 is flipped away from the Mg^2+ ^ion, a conformation resembling the "state 1" as described for the GDP-bound conformation of the wild-type protein [6]. However, loop L2 is weakly defined and displays essentially no interpretable electron density for residues 30-32, so that these residues were omitted for refinement. The conformation of residues 60-74 and 98-108 (switch II and residues interacting with it) resemble more the conformation in the unphotolyzed, caged GTP-bound protein (crystal C4) than in wild-type H-Ras•GDP. This shows that the GTP hydrolysis reaction does not depend on large-scale conformational changes in the switch regions of H-Ras as observed in solution or as deduced from comparisons of GDP- and GTP-bound crystal structures. Geometric restrictions by the crystal lattice might not allow rearrangements in the crystal to take place to the same extent as in solution, even though complete GTP hydrolysis occurs with comparable half-times [7]. Therefore, residues involved in large structural changes get trapped in multiple conformations representing different local minima in the energy landscape, which results in uninterpretable electron density for those regions.

### Conformational dynamics in H-Ras p21 at very high resolution

**Figure 3:**
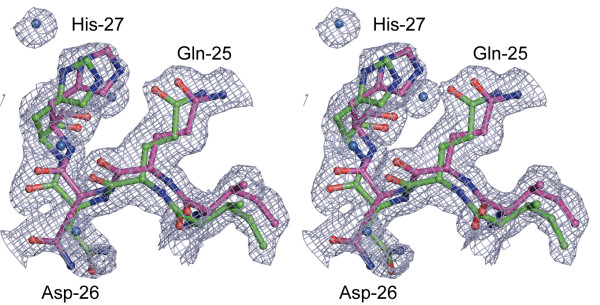
**Superposition of sub-conformations**. Stereo representation of small differences between the two sub-conformations of residues Ile24-His27 in p21(mod)•R-caged GTP (green and magenta sticks, respectively). The displayed 2F_obs_-F_calc _omit electron density map was calculated by the randomized omit map procedure after refinement of the model structure with the program REFMAC5 [16] (0.9 σ cutoff level, displayed with the program Pymol [24]).

**Figure 4:**
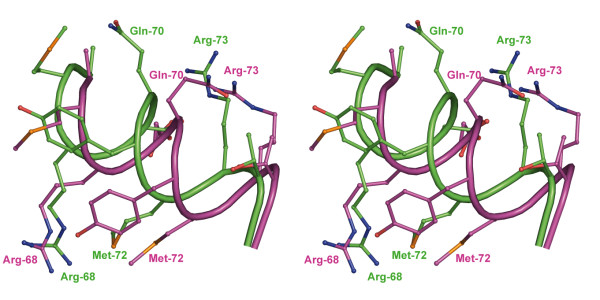
**Stereo representation of conformational heterogeneities within helix α2**. Shown are the two different sub-conformations of residues 66-75 in p21(mod)•R-caged GTP (green and magenta sticks, respectively).

**Figure 5:**
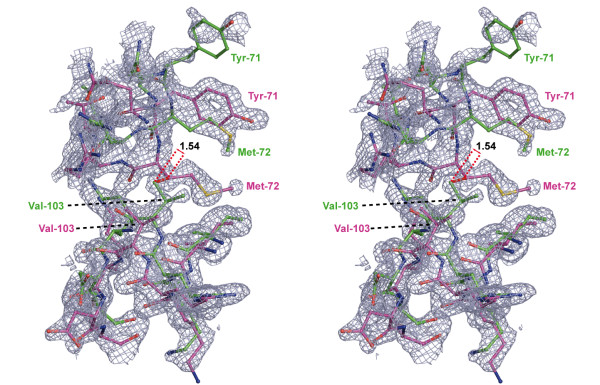
**Stereo representation of conformational heterogeneities in vicinity of the contact region of Met72 and Val103**. Shown are the two sub-conformations of residues 70-74 and residues 100-106 in p21(mod)•R-caged GTP. The displayed 2F_obs_-F_calc _omit electron density map was calculated by the randomized omit map procedure after refinement of the model structure with the program REFMAC5 [16] (0.65 σ cutoff level, displayed with the program PyMOL [24]).

The electron density in structures of H-Ras p21 at very high resolutions (crystals C2-C7) could only be adequately interpreted by assuming alternative conformations not only for the flexible switch regions, but also for most of the remaining protein chain. Optimal results were obtained by the 'two-chain-isotropic-refinement' strategy (see Materials and Methods), in which the whole protein chain is described with two conformations. The deviation between both chains was only marginal in the GDP-bound crystal C2, but the high resolution of 1.0 Å still required both chains for a reasonable fit to the electron density. As expected, the most significant deviations (typically less than 1.5 Å) between both alternative conformations were found in the flexible switch regions and residues which are in direct contact with those (aa 59-63, 99-109, 121-123, and 132-138). An even higher conformational flexibility was observed in crystal structures of H-Ras in complex with caged GTP and of crystals derived from those by *in crystallo *reactions (crystals C3-C7). This is exemplified in Figure 3: Even regions which are not involved in direct contact with the active site and are normally not considered to be flexible show clearly deviating alternative conformations. Residues 67-74 display one alternative conformation which is almost identical to structures of the wild-type protein in complex with GTP nucleotide analogues (PDB entry codes 5P21, 1QRA, 1CTQ and 1GNR). In contrast, the second conformation deviates significantly from the first one, both in side-chain and main-chain conformation (Figures 4 and 5). Despite of a higher noise level in this region, the conformation of the whole main-chain and most side-chains could unambiguously be interpreted, and a significant reduction of the free R-factor indicated that the second chain is necessary for a comprehensive interpretation of the observed electron density. Remarkably, in the contact region of Met72 and Val103 (Figure 5), both residues show clear electron density for their side-chains, with a minimum distance of only 1.55 Å. This distance is too small to be physically possible with only one conformation present in the protein. The conformational heterogeneity in residues 66-74 coincides with heterogeneity of the contacting residues Glu98 - Asp108, which were also interpreted by two significantly deviating main-chain conformations (Figure 5).

The high data quality, which allowed an application of the 'two-chain-isotropic-refinement' strategy, for the first time provides detailed information about the conformational dynamics of H-Ras p21 in its crystalline state. The high resolution, combined with the fortunate situation that most residues of the analyzed structures seemed to occupy not more than two highly populated subconformations, allowed us to create a model which shows H-Ras p21 in two alternative overall protein folds. However, it should be noted that the presence of additional, weaker populated subconformations is likely, and that further investigations will have to prove to what extent the two observed subconformations represent functionally relevant states. It is worth mentioning that high *B*-factors and weak or very noisy electron density distributions of regions contacting the flexible loops are observed in almost all structures of Ras-like GTPases [8]. Interestingly, the conformation of residues Glu98 - Asp108 of GTP-bound H-Ras p21 (PDB entry 1CTQ[28]) represent an average of the two alternative conformations found in p21(mod) in complex with R-caged GTP. The wild-type protein therefore appears to have similar alternative conformations, which would only become interpretable if data with very high resolution were available.

### Different hydrolysis rates for R- and S-caged GTP

Crystals containing S-caged GTP showed a significantly higher hydrolysis rate for non-photolyzed S-caged GTP to caged P_i _and GDP (80% hydrolysis within 100 days) than crystals containing R-caged GTP (22% hydrolysis within 100 days), as revealed by HPLC analysis. Such different reaction rates have also been observed for the wild-type protein (Scheidig et al. [29]), but due to a low resolution (2.5 Å for the S-caged GTP dataset), an explanation based on structural details was not possible. Since the hydrolysis occurs with a mechanism similar to physiologic GTP to GDP hydrolysis, this finding might help understanding the requirements for efficient hydrolysis in general, even though the caged nucleotides are artificial. An influence of varying crystallization parameters can be ruled out, as complexes with both diastereomers were obtained in identical crystallization setups, and had comparable size, lattice parameter and diffraction power. In the present study, resolutions of 1.05 Å for the R-caged GTP dataset (C4) and 1.24 Å for the S-caged GTP dataset (C3) were available. This allows a detailed insight into the structural background for this phenomenon.

**Figure 6:**
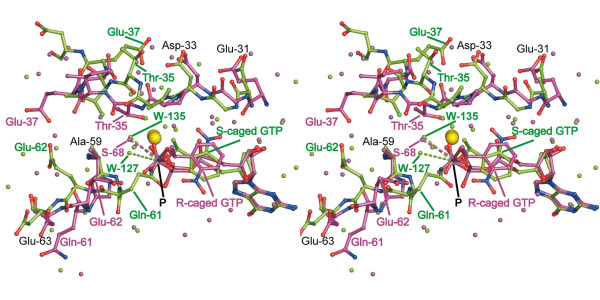
**Region with large structural heterogeneity**. Stereo representation of a region with large differences between p21(mod)•R-caged GTP (magenta sticks) and p21(mod)•S-caged GTP (green sticks). Shown are residues 31-38, residues 59-63, the nucleotide with the coordinated Mg^2+ ^ion (yellow sphere), and surrounding water molecules. The complete side chain of the NBD-modified residue Cys-32 was omitted for a better graphical representation. The hydrolytically active water molecules are indicated by dashed lines to the γ-phosphorous atom of the nucleotide.

We propose that a different positioning of water molecules surrounding the γ-phosphate of the nucleotide has an important role in the accelerated hydrolysis of S-caged GTP to GDP and caged P_i _compared with R-caged GTP hydrolysis: The most substantial difference between both structures is an approximately 180°rotation of the cage group of the nucleotide (Figure 6). While in crystals containing R-caged GTP the nitro group of caged GTP is oriented away from loop L2, it orients towards loop L2 residues 31-33 in p21(mod) complexed with S-caged GTP, forming a hydrogen-bond with Asp33. Although this interaction induces only small differences in residues 30-34, the adjacent region of Thr35 to Asp38 is significantly affected and displays strongly deviating conformations in the two structures. In this way, contacting residues Gly60-Glu63 are also reordered, so that only in p21(mod) complexed with S-caged GTP the sidechain of Gln61 is oriented towards the nucleotide (Figure 6). Gln61 can position and activate water molecule W127, which together with the carbonyl-oxygen of Pro34 activates in a concerted manner the hydrolytically active water molecule W135. In contrast, only one ordered water molecule was observed in p21(mod) complexed with R-caged GTP, which was not coordinated by Gln61 (Figure 6). This explains the increased hydrolysis rate of S-caged GTP and provides an interesting insight into the requirements for efficient hydrolysis. In addition to a more optimal activation of the hydrolytically active water molecule, the conformation of the switch I region in crystals containing S-caged GTP resembles the conformation in wild-type H-Ras complexed with GDP (PDB entry code 4Q21; [17]). Like in the wild-type structure, Thr35 undergoes no direct interactions with the Mg^2+ ^ion or the nucleotide. This might additionally reduce the energy barrier for the decay of caged GTP to GDP and caged P_i_.

### A second Mg^2+ ^binding site at the nucleotide might also be relevant in the wild-type protein

**Figure 7:**
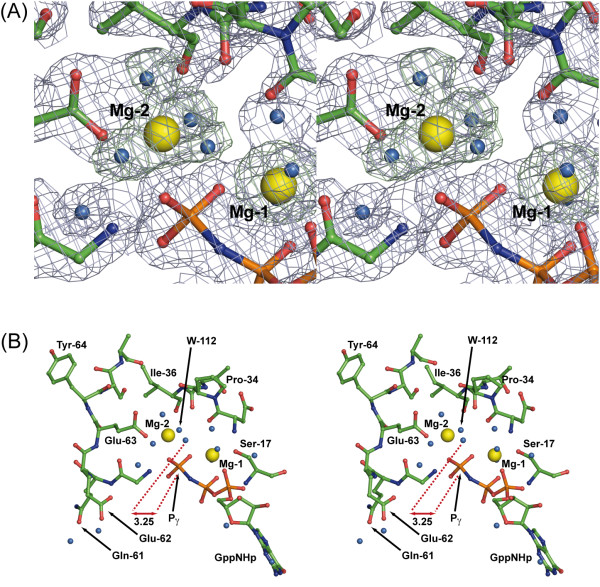
**Stereo representation of the second Mg**^**2+ **^**ion binding site within the active center of p21(mod)•GppNHp**. (**A**) The displayed omit electron density map was calculated with the program REFMAC5 [16] by the randomized omit map procedure after removal of both Mg^2+ ^ions and the coordinating water molecules around the second Mg^2+ ^ion (in blue, 2F_obs_-F_calc _map with 2.0 σ cutoff level and in green, F_obs_-F_calc _map with 2.5 σ cutoff level; the figure was produced with the program PyMOL [24]). (**B**) Shown are residues 17, 33-36 and 60-66 (green sticks), the nucleotide which is coordinated to the two Mg^2+ ^ions Mg-1 and Mg-2 (yellow spheres), and surrounding water molecules (cyan spheres). Both (A) and (B) show identical orientations of the active site with different zoom factors.

The structure of p21(mod)•GppNHp displays several features which were not observed in wild-type H-Ras p21 bound to GTP analogues. The most important of these is a second well-defined metal ion coordinating the γ-phosphate. This metal ion forms contacts with the nucleotide and one oxygen of the carboxyl-group of Glu63, and additional water-mediated contacts with the main chain oxygen atoms of Asp33, Pro34, and Thr35. Due to an octahedral coordination sphere and observed coordination distances between 2.03 Å and 2.13 Å, this metal ion was interpreted as a second Mg^2+ ^ion (Mg-2) (Figure 7). Both Mg^2+ ^ions in p21(mod)•GppNHp are interpreted with 100% occupancy and are refined to similar individual *B*-factors of 21.9 Å^2 ^(Mg-1) and 28.5 Å^2 ^(Mg-2), respectively. All water molecules directly coordinating to both Mg^2+ ^ions also refine to similar *B*-factors (23.4-27.6 Å^2 ^and 28.3-31.2 Å^2 ^for water molecules coordinating MG-1 and MG-2, respectively). Binding of the second Mg^2+ ^ion induces significant differences in the protein conformation in proximity to the nucleotide. Pro34 in p21(mod)•GppNHp is shifted away from the nucleotide, since a position similar as in wild-type H-Ras complexed with GppNHp would overlap with the position of the second Mg^2+ ^ion. Probably due to this distortion in the main chain of the switch I region, the side-chain hydroxyl group of Thr35 is not coordinated to the first Mg^2+ ^ion, instead forming a hydrogen bond to the side chain of Asp33. The electron density for residues 60-63 in the switch II region is well defined in p21(mod)•GppNHp due to the anchored Glu63 and a turn motif with a hydrogen bond between the carbonyl oxygen of Gly60 and the amide nitrogen of Glu63. Therefore, the conformation of loop L4 deviates significantly from known structures of the wild-type protein. In consistence with a strong interaction of Glu63 with the well-ordered MG-2 atom, the *B*-factor of 36.6 Å^2 ^of the contacting Glu63 carboxyl oxygen is significantly smaller than *B*-factors of other atoms of surrounding switch II residues, which are in the range of ~45 Å^2^.

The large differences between p21(mod)•GppNHp and p21(wt)•GppNHp are quite unexpected. The mutation C118S does not induce significant deviations between the modified protein and the wild-type protein in the vicinity of Ser118. Therefore, the introduced fluorophore is the only artificial group with the potential to significantly affect the protein structure. However, the fluorophore is located at a similar position and points to the same direction as was observed for Tyr32 in the crystal structure of the wild-type protein complexed with GppNHp [18]. Furthermore, it does not undergo any strong interactions with other groups and it is located in relatively weak electron density in the solvent space of the crystal. Strong stacking interactions with other residues like in p21(mod) complexed with R/S-caged GTP (see above), which are responsible for a significant distortion in some regions of these structures, are not observed in p21(mod)•GppNHp due to the lack of a cage group at the nucleotide.

The question arises why such large deviations to the wild-type protein occur, whereas the GDP-bound state is almost identical to the wild-type protein (see above). In most known structures of H-Ras p21 in complex with GTP, GppNHp or GppCH_2_p, Tyr32 interacts with the γ-phosphate of the nucleotide from a neighboring molecule (PDB entry codes 1CTQ, 1QRA and 5P21) or from the same molecule (PDB entry code 6Q21, [17]). Since H-Ras does not form dimers in solution, it is obvious that the intermolecular interaction in the wild-type protein is a crystallization artifact. By exchanging Tyr32 with a fluorescent group in p21(mod), the capability of the protein to form this artificial crystal contact is eliminated. As a consequence, other interactions with the γ-phosphate can be established.

In a crystal structure of RalA•GppNHp, which is another GTPase of the Ras subfamily, Nicely et al. [30] also describe a second Mg^2+ ^ion which is coordinated to the γ-phosphate group of the nucleotide. Despite of different conformations of the switch regions compared to H-Ras, the nucleotide is almost identically positioned, and the observed second Mg^2+ ^ion in RalA binds at a similar (though not identical) position as found in p21(mod)•GppNHp. Interestingly, only one of two molecules in the asymmetric unit of RalA contain a second Mg^2+ ^ion, probably due to an uncommon fold of the switch I region of one of the molecules, resulting in a favorable situation for the binding of a second Mg^2+ ^ion [30]. In another context, a recent study of the *S. flexneri *effector protein IpgB2 in complex with the Rho GTPase RhoA demonstrates the presence of two alternative Mg^2+ ^binding sites in complexes of wild-type RhoA•GDP with IpgB2 [31]. Different treatment of crystals from identical crystallization conditions lead to population or depopulation of this secondary binding site. Although such a secondary Mg^2+ ^binding site was not described before, almost all related Rho GTPases present cavities which would allow similar secondary Mg^2+ ^binding without significant conformational rearrangements, indicating a potential relevance of this site for the function of the GTPase.

Although binding of Mg^2+ ^to secondary binding sites might strongly depend on crystal contacts and crystallization conditions, one should note that crystallization conditions are essentially always artificial. This is particularly true for H-Ras p21, where structures of the wild-type protein present artificial intra-molecular salt-bridges with the nucleotide binding site, which renders derived information on the metal ion coordination of the nucleotide questionable without independent experiments. Even though the finding of a second Mg^2+ ^ion bound to the nucleotide in p21(mod)•GppNHp does not necessarily prove that this interaction is relevant *in vivo*, it does unambiguously show that such an interaction is at least energetically possible. We showed that the binding of a second Mg^2+ ^ion to the γ-phosphate of the nucleotide is strong enough to display a well-defined octahedral coordination sphere, and that p21(mod) in complex with GDP forms a tertiary structure essentially identical to the wild-type protein. So what can the second Mg^2+ ^ion in the modified protein tell us about the mechanism of GTP hydrolysis, and how probable is a similar mechanism in the wild-type protein?

There is evidence from combined quantum mechanical and molecular mechanical (QM/MM) calculations by Klahn and coworkers [32] that binding of a second cation to H-Ras might be involved in catalysis by fixing one water molecule close to the γ-phosphate. The presence of a second cation would explain several experimental findings, such as the protonation state of Ras-bound GTP at physiological pH values [33] and an increased intrinsic GTPase activity of p21(Q61E) [34]. The general tendency of the γ-phosphate to coordinate to Mg^2+ ^is also evident from studies in aqueous solution, where GTP binds Mg^2+ ^in a tridentate manner at pH 7.5 [35]. In agreement with the calculations of Klahn *et al. *[32], the second Mg^2+ ^ion in p21(mod)•GppNHp fixes a water molecule (W122) at a favorable distance (3.3 Å) for nucleophilic attack of the γ-phosphorous atom (Figure 7). This is significantly closer than the distance of the hydrolytically active water molecule to the γ-phosphorous atom in structures of the wild-type protein (3.69 Å in PDB entry 5P21, 3.63 Å in 1CTQ, 4.74 Å in 1GNR, and 3.69 Å in 1QRA). In this way, the second Mg^2+ ^ion coordinating the γ-phosphate in p21(mod)•GppNHp might activate the hydrolytically active water molecule, as proposed by Klahn *et al. *[32] for the wild-type protein.

It is plausible that the reason for the binding of a second cation in p21(mod)•GppNHp might be the elimination of artificial intermolecular interactions of Tyr32 and not a direct effect of the weakly coordinated fluorophore attached to Cys32 itself. According to these considerations, the binding of a second Mg^2+ ^ion might be of relevance for a more detailed understanding of the wild-type protein. It has to be clarified in further experiments if the wild-type protein indeed is able to bind Mg^2+ ^in a similar manner as in p21(mod)•GppNHp, probably only via transient interactions, and how relevant these interactions are for the catalytic mechanism of H-Ras p21 and related GTPases.

## Conclusions

A fluorescent form of H-Ras p21 ("p21(mod)") was designed to analyze the protein's conformational dynamics by kinetic crystallography experiments. X-ray diffraction experiments on p21(mod) complexed with different nucleotides at very high resolution allowed to distinguish two discrete alternative conformations for the whole protein chain. Similar alternative conformations are clearly also present in the wild-type protein, but only become interpretable with very high data quality.

Even though the modified protein has a reduced GTPase activity, the structure in complex with GDP is highly similar to the wild-type, proving that the introduced modifications (Y32C, C118S, C32-IANBD-amide) do not necessarily induce an artificial protein fold. Since no structure of wild-type H-Ras p21 in complex with GDP with more than 2 Å resolution is currently available, the structure of p21(mod)•GDP at 1.0 Å represents a high-resolution model structure for H-Ras p21 in the GDP-bound state.

Flash photolysis and subsequent GTP hydrolysis did not induce substantial changes of the protein structure, even though the electron density in vicinity of the nucleotide displayed complete hydrolysis to GDP. It was a general finding with only few exceptions that regions undergoing large rearrangements upon *in crystallo *reactions became disordered and no longer occupied well-defined positions after the rearrangement. This shows that geometrical restrictions induced by the crystal lattice, even though excluding conformational changes upon GTP hydrolysis as observed in solution, do not necessarily hinder the enzymatic reaction.

We could explain the significantly faster decay of S-caged GTP to GDP and caged P_i _compared to R-caged GTP by a stronger activation of the hydrolytically active water molecule due to different conformations of the switch I regions of p21(mod) induced by different interactions with the nitro groups of R/S-caged GTP.

One of the most significant differences to known wild-type structures was the finding of a second Mg^2+ ^ion in the active site of p21(mod)•GppNHp. The second Mg^2+ ^ion positions the hydrolytically active water molecule at a significantly smaller distance to the γ-phosphorous atom than in wild-type structures in complex with GTP-analogues, which might have an important role in activation of that water molecule for a nucleophilic attack [32]. In this respect, a secondary Mg^2+ ^binding site might also be relevant for the GTP hydrolysis pathway of wild-type H-Ras p21. The reason why such a secondary Mg^2+ ^binding sites has not been described before in crystal structures of H-Ras p21 might be artificial intramolecular contacts of Tyr32 with the nucleotide, which are eliminated in p21(mod) by the fluorophore attached to Cys32. Secondary Mg^2+ ^binding sites were also observed in other GTPases like RalA or RhoA [30,31]. These additional Mg^2+ ^binding sites might play a more substantial role for the intrinsic activity of GTPases than generally appreciated.

## Authors' contributions

BUK participated in the design of the study, carried out all experiments in molecular biology, protein chemistry, kinetic crystallography and structure refinement. AJS designed the study, participated in experimental design and structure refinement. The manuscript was drafted by BUK and AJS. All authors read and approved the manuscript.
